# Data on characterization and electrochemical analysis of zinc oxide and tungsten trioxide as counter electrodes for electrochromic devices

**DOI:** 10.1016/j.dib.2020.105891

**Published:** 2020-06-20

**Authors:** Nguyen Sy Pham, Young Hun Seo, Eunji Park, Thao Dang Duy Nguyen, Ik-Soo Shin

**Affiliations:** a Department of ICMC Convergence Technology, Soongsil University, Seoul 06987, Republic of Korea; b Department of Chemistry, Soongsil University, Seoul 06987, Republic of Korea

**Keywords:** Zinc oxide nanowire, Tungsten trioxide, Electrodeposition, Charge storage capacity, Electrochromic device

## Abstract

The data presented in this article are related to a research paper entitled “**Implementation of High-Performance Electrochromic Device Based on All-Solution-Fabricated Prussian Blue and Tungsten Trioxide Thin Film**”[1]. Zinc oxide nanowire (ZnNW) and tungsten trioxide (WO_3_) were fabricated by different electrodeposition methods and characterized by X-ray diffraction (XRD), X-ray photoelectron spectroscopy (XPS), scanning electron microscope (SEM) and used as counter electrodes. The electrochromic (EC) properties of these devices were characterized using optoelectronic analysis during electrochemical applications.

Specifications table

**Table:** 

Subject	Electrochemistry
Specific subject area	Electrochromic devices
Type of data	Figures
How data were acquired	XRD, XPS, SEM, CHI660D electrochemical workstation, UV–visible spectrometer (UV–vis)
Data format	Raw and analyzed
Parameters for data collection	XRD results give an insight into the crystallinity of the ZnNW and amorphous WO_3_ films. The elemental, morphological, and optical analysis of three kinds of WO_3_ films were carried out using XPS, SEM, and UV–vis spectroscopy, respectively.
	The electrochemical properties of WO_3_-TPA film were analyzed using CV measurements in a customized three-electrode configuration, wherein the WO_3_-deposited ITO, Pt wire, and Ag/AgCl (saturated KCl) were used as the working, counter and reference electrode, respectively.
Description of data collection	Preparation of ZnNW and WO_3_ films on ITO glass. Characterization and electrochemical measurements of the ZnNW and WO_3_ films.
Data source location	Electrochemistry Lab, Soongsil University, Korea.
Data accessibility	Data is with the article
Related research article	Nguyen Sy Pham, Young Hun Seo, Eunji Park, Thao Dang Duy Nguyen, and Ik-Soo Shin, Implementation of High-Performance Electrochromic Device Based on All-Solution-Fabricated Prussian Blue and Tungsten Trioxide Thin Film. Electrochimica Acta, DOI:10.1016/j.electacta.2020.136446

## Value of the data

• In electrochromic devices (ECDs), redox half-reactions occur simultaneously at each electrode. For example, while the EC layer at the working electrode (WE) undergoes an oxidation half-reaction, its counter electrode (CE) undergoes the opposite reduction half-reaction at the same time [2,3]. Therefore, a more sluggish half-reaction at the CE will hamper the half-reaction of the EC layer at the WE. If the charge storage capacity at the CE is smaller than that of the EC layer at the WE, the EC performance of the device will be degraded [1]. For these reasons, CE plays a crucial role in the performance of ECDs. Electrodeposition stands out as a promising fabrication technique due to its simplicity, relatively low costs, and the high electroactive surface area of films or nanostructures formed via this method [4]. The data in this article gives the characterization, electrochemical and optical analysis of ZnNW and WO_3_ films as CEs fabricated by various types of electrodeposition methods.
• Researchers who are interested in the influence of electrodeposition methods on film fabrication and CEs for ECDs can benefit from these data.
• The crystallinity of ZnNW and amorphous WO_3_ films were confirmed using XRD. The elemental, morphological, and optical analysis of three kinds of WO_3_ films were investigated using XPS, SEM, and UV–vis, respectively, which shows the significant impact of electrodeposition methods on film fabrication and electrochromic performance. CV results display the electrochemical behavior of the WO_3_-TPA film at scan rates of 20–100 mV s^−1^ at 2nd and 2000th cycle, indicating its fast and reversible redox properties.
• The data can be further used as base values and for comparison to other scientific reports. Additionally, the data presents evidence that the WO_3_ film fabricated by the TPA electrodeposition method can be used as a potentially efficient counter electrode for ECDs in future studies.

## 1 Data description

**Fig. 1:**
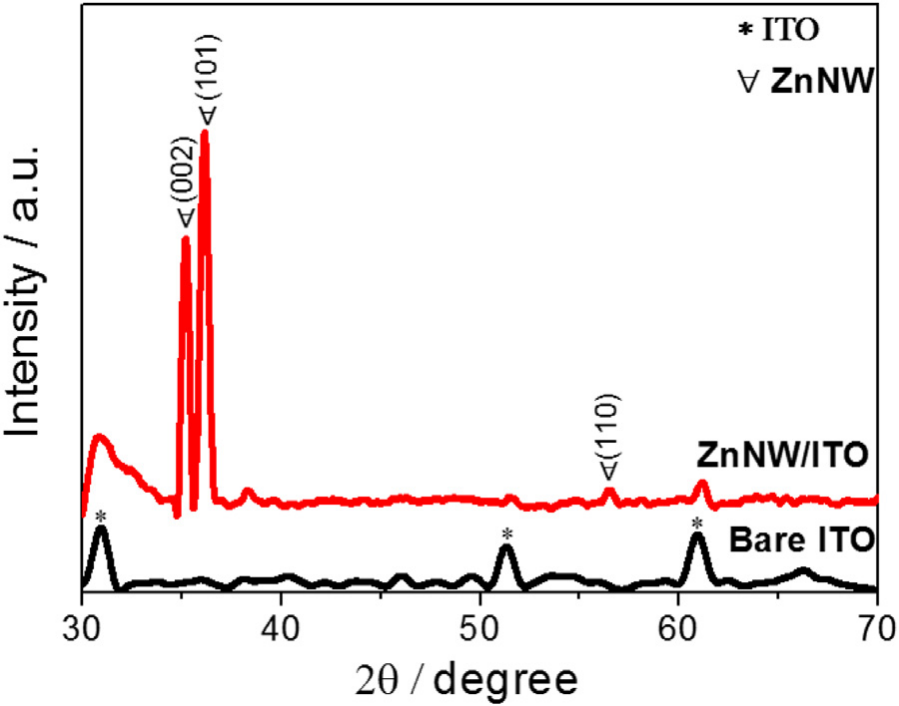
XRD pattern of ZnNW on ITO glass.

**Fig. 2:**
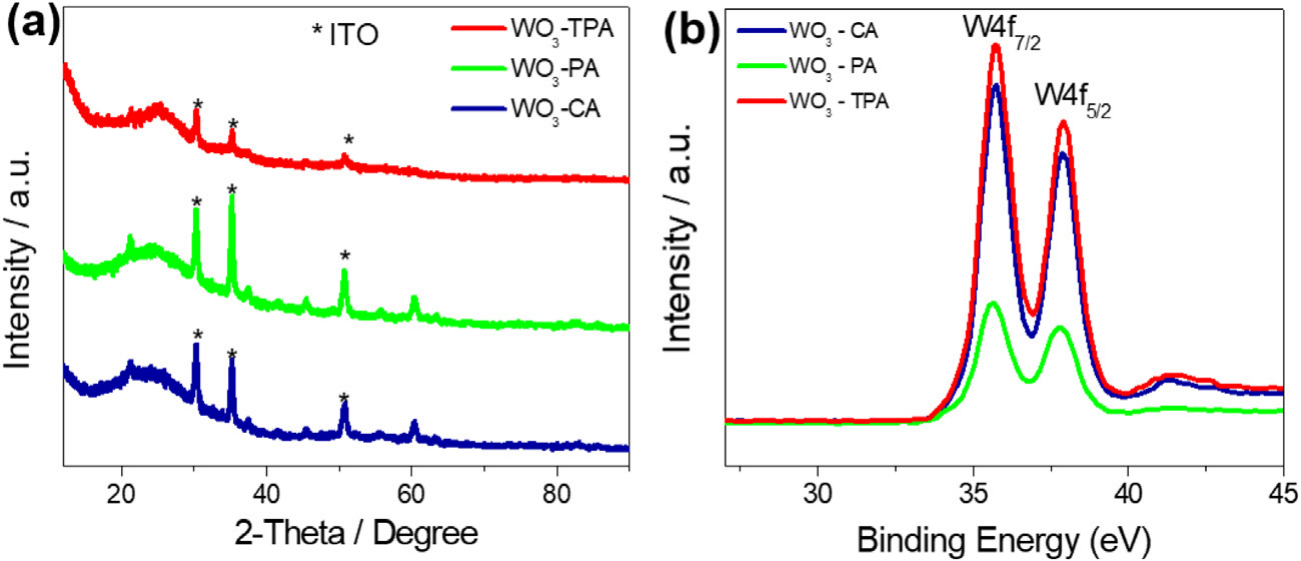
(a) XRD pattern of the WO_3_ film prepared by TPA, CA and PA electrodeposition, (b) XPS survey spectra of the W4f peak of the WO_3_ films prepared by TPA, CA and PA electrodeposition.

**Fig. 3:**
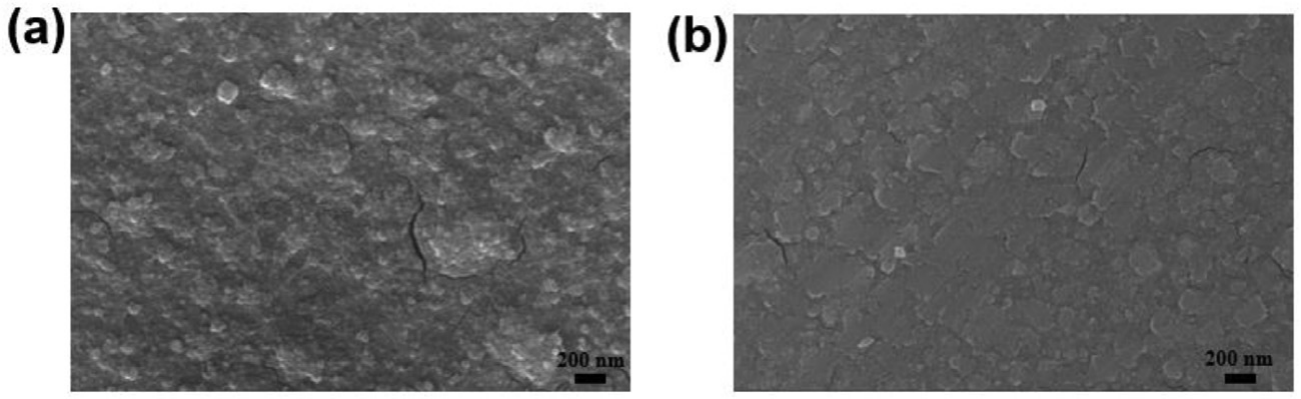
SEM images of the WO_3_ film prepared by (a) CA and (b) PA.

**Fig. 4:**
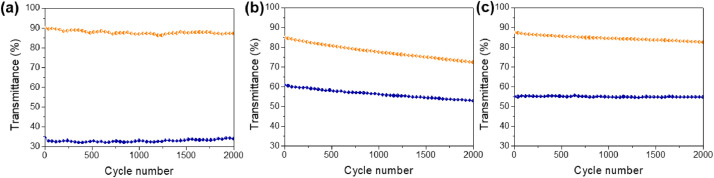
(a) In situ optical responses (λ = 633 nm) of the WO_3_-TPA under the application of stepwise potential (between **−**0.5 and +0.3 V for 4 s per step) for 2000 operational cycles. (b) In situ optical responses (λ = 633 nm) of the WO_3_-PA under the application of stepwise potential (between **−**0.5 and +0.3 V for 4 s per step) for 2000 operational cycles. (c) In situ optical responses (λ = 633 nm) of the WO_3_—CA under application of the stepwise potential (between **−**0.5 and +0.3 V for 4 s per step) for 2000 operational cycles.

**Fig. 5:**
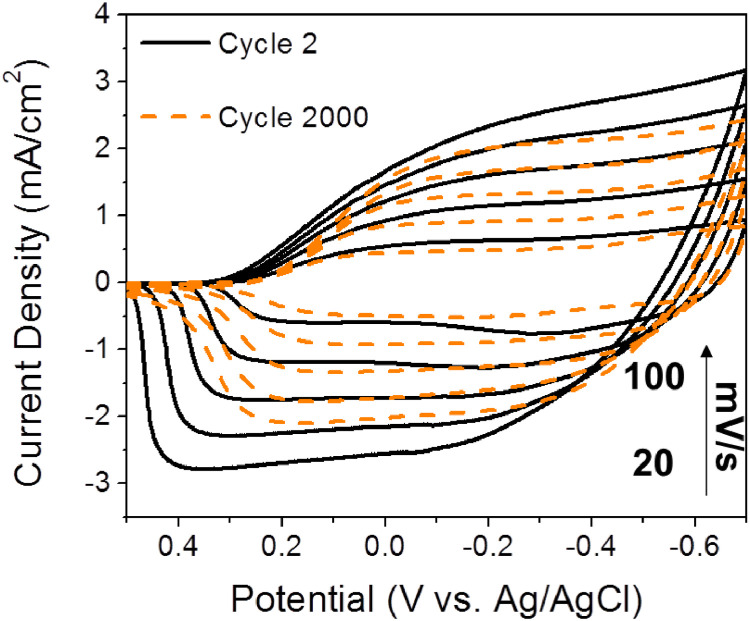
Cyclic voltammograms of WO_3_ film deposited by TPA method on ITO glass acting as WE in 0.1 M K_2_SO_4_ at different scan rates in the potential range of −0.7 to 0.5 V vs Ag/AgCl at 2nd cycle (solid line) and 2000th cycle (dash line).

Fig. 1 shows the characterization of ZnNW by the XRD pattern. Also, the characterization of three kinds of WO_3_ films was conducted with XRD and XPS measurements in Fig. 2. Fig. 3 shows the surface of WO_3_—CA and WO_3_-PA. As displayed in Fig. 4, the transmittance as a function of the cycle for different WO_3_ films was characterized using UV–vis with a customized three-electrode configuration, wherein the WO_3_-deposited ITO, Pt wire and Ag/AgCl (saturated KCl) were used as the working, counter and reference electrode, respectively. Fig. 5 shows the CV of WO_3_-TPA. The raw data for this work are presented in the Supplementary section.

## 2 Experimental design, materials, and methods

### 2.1 Preparation of substate

The ITO (10 Ω/sq, commercially purchased) deposited glass substrate was cut into pieces of 0.8 cm x 0.9 cm, then ultrasonically washed with isopropyl alcohol, acetone, and deionized water for 15 min in that order before being dried in air at 60 °C. Impurities and oily substances on the ITO surface can have adverse effects on the electrodeposition of the materials. As a result, these films are not firmly adhered to the ITO glass, and hence are liable to flake off during storage. To handle this problem, UV-ozone cleaning was used to clean the ITO glass for 45 min to remove contaminants and improve the adhesion between the ITO glass and the thin film material.

### 2.2 Deposition of ZnO nanowire on ITO glass

The electrodeposition method was selected because it is a low-cost, environmentally friendly process that is scalable on large-area substrates. Moreover, using this technique, nanostructures can be deposited and used without any annealing treatment, which is highly beneficial for the preparation of ZnNW on ITO glass [5]. The ZnNW electrodeposition was carried out in a standard three-electrode system in which a platinum wire and a Saturated Calomel Electrode (SCE) were used as the counter and reference electrode, respectively. The process consists of two steps as follows: The ZnO thin films (buffer layers) were deposited in an electrolyte containing 5 mM ZnCl_2_ and 0.1 M KCl at a potential of −1 V at room temperature. Subsequently, ZnNW was deposited from an electrolyte including 0.5 mM ZnCl_2_ and 0.1 mM KCl at a constant potential of −1 V at 70 °C. For all experiments, electrolytes were saturated by intensive molecular oxygen bubbling for 1 h prior to ZnNW electrodeposition. The ZnNW on ITO glass was analyzed by XRD.

### 2.3 Deposition of WO_3_ on ITO glass

The WO_3_ film was synthesized via the triple pulse amperometry (TPA) method [6]. This method is the best electrodeposition method for WO_3_ film fabrication with near-ideal optical modulation, fast switching speed, high coloration efficiency, large charge capacity, and excellent cycling stability. The WO_3_ film was synthesized in the electrolyte prepared by dissolving 0.082 g of Na_2_WO_4_•2H_2_O in 20 ml of deionized water, 0.16 ml of perchloric acid, and 52 μl of H_2_O_2_. The pulsed electrodeposition with 1.1 s interval time between each pulse was performed with a CHI660D electrochemical workstation at room temperature using a conventional three-electrode system. A series of two square waveform pulses were applied to the cell in each deposition cycle as follows: one pulse at −0.7 V for 0.1 s and the other at 0 V for 0.1 s. A pause of 1.1 s was added in between the cycles. The entire deposition process consisted of 2000 cycles, amounting to a deposition time of 2600s. At the end of the deposition process, the films were thoroughly rinsed with water and dried in air. The WO_3_ film (WO_3_—CA) was synthesized using continuous electrodeposition at −0.7 V while the WO_3_ film (WO_3_-PA) was synthesized using similar conditions to the synthesis of WO_3_-TPA without a pause of 1.1 s for comparison. The WO_3_ films on ITO were analyzed by XRD and XPS. Electrochemical measurements were conducted in a three-electrode electrochemical cell in which WO_3_-deposited ITO, Ag/AgCl, and Pt wire were used as the working, reference, and counter electrode, respectively.

## Declaration of Competing Interest

The authors declare that they have no known competing financial interests or personal relationships that could have appeared to influence the work reported in this paper.

## Appendix Supplementary materials

Image, application 1
