# Supplementary figures and images for: Data on characterization and electrochemical analysis of zinc oxide and tungsten trioxide as counter electrodes for electrochromic devices
Source: Data Brief. 2020 Jun 20;31:105891. doi: 10.1016/j.dib.2020.105891 (PMC7326711; doi:10.1016/j.dib.2020.105891)

## Slide 1
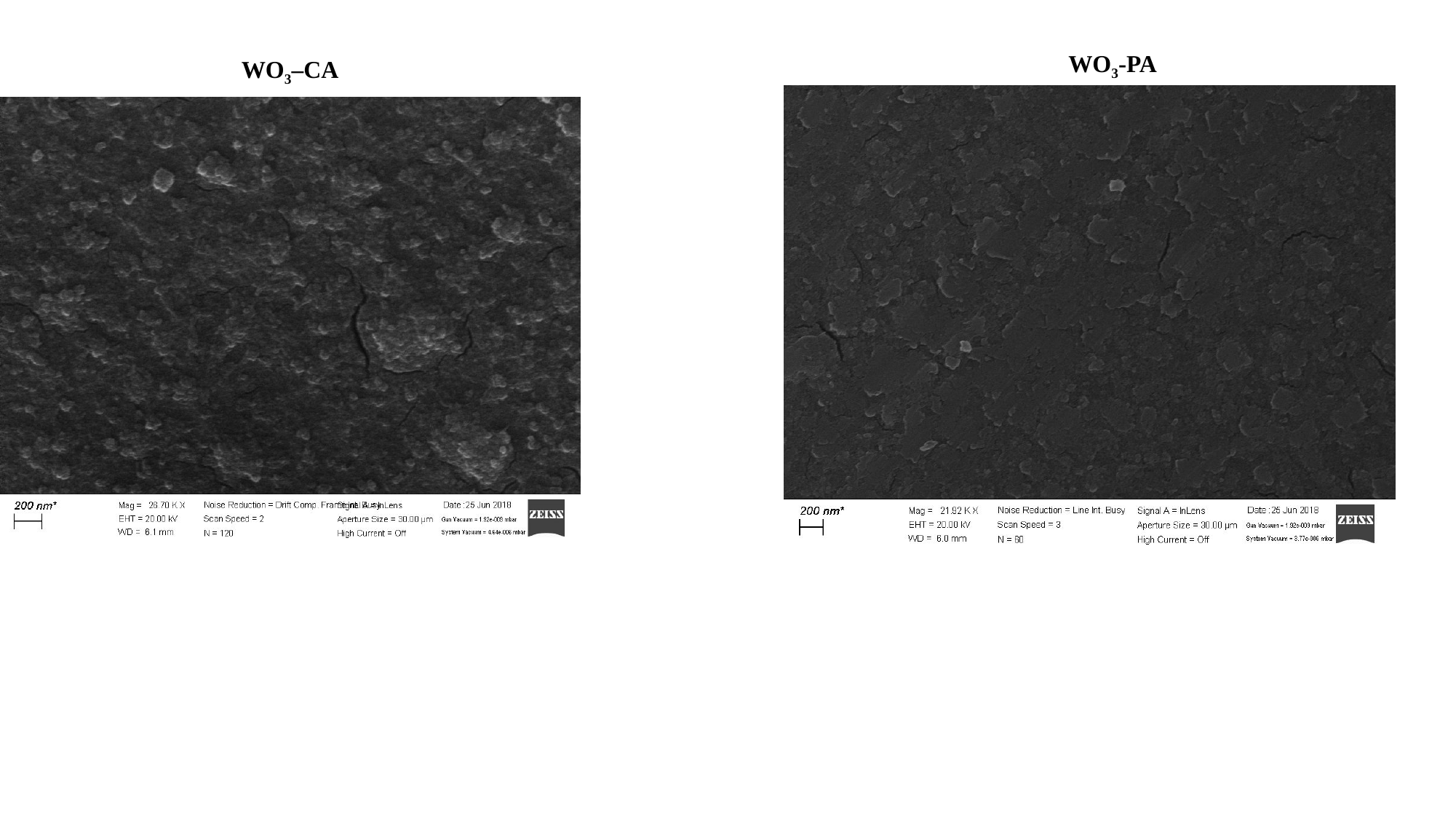

WO3-PA
WO3–CA

Supplement: Supplementary file 1 [file mmc1.zip › Raw data/Figure 3/SEM of WO3-CA and WO3-PA.pptx]
